# Cutting-edge transformer-based deep learning for Parkinson's disease diagnosing using multimodal hand-drawing and handwriting analysis

**DOI:** 10.3934/Neuroscience.2026015

**Published:** 2026-06-16

**Authors:** Theyazn H.H Aldhyani

**Affiliations:** Applied College, King Faisal University, P.O. Box 400, Al-Ahsa 31982, Saudi Arabia

**Keywords:** Parkinson's diagnosis, prediction, transformers, deep learning, artificial intelligence

## Abstract

Parkinson's disease (PD) is a significant mental health condition, and patients greatly benefit from prompt diagnosis and treatment if the disease is identified early. One powerful method for diagnosing PD at an early stage is the analysis of hand-drawing and handwriting samples from individuals with PD. The novelty of this research lies in developing a handwriting and hand-drawing Parkinson's Disease (HwdPD) framework for detecting PD. This framework utilizes hand-drawing and Arabic handwriting samples, which have been observed to be effective in detecting PD. Based on VGG19 and transformer, the proposed framework was tested using a real standard dataset containing 63 hand-drawn images, namely spiral, wave, and ellipse samples, with 30 samples from PD and 33 from healthy patients. The dataset also contains Arabic handwriting samples, namely “eight” and hello (“لو”). These images were processed by using augmentation to enhance the performance of the HwdPD framework. This enhancement of the image area was fed to the classification algorithm (transformer ViT-B16 and VGG19). ViT-B16 scored high accuracy, with 100% in spiral, wave, and ellipse images. In handwriting samples (“eight”), the system successfully achieved a high percentage of 100%. This system emphasizes the potential to improve diagnostic accuracy and assist clinical decision-making by evaluating its performance on these datasets. The HwdPD framework demonstrates potential for identifying PD biomarkers, which may lead to improved diagnostics.

## 1. Introduction

**Figure 1.**
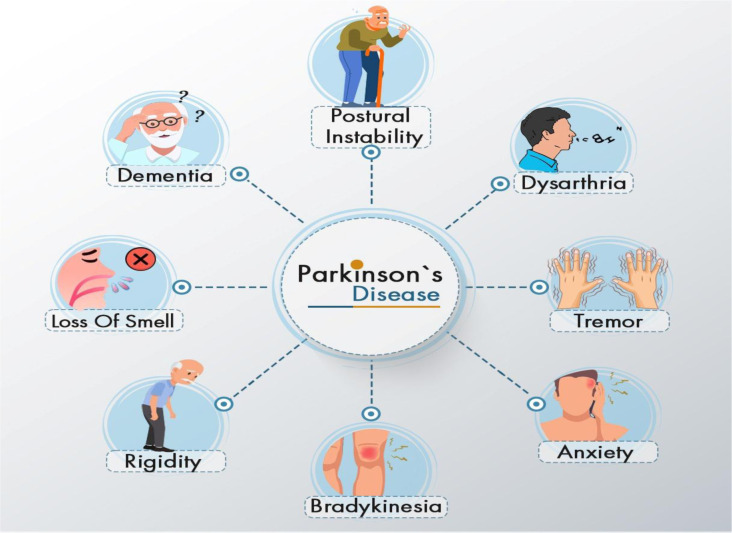
PD symptoms.

Parkinson's disease (PD) is a mental disorder characterized by the degeneration of a specific region of the brain, resulting in progressively worsening symptoms. This disorder is most recognized for its impact on muscular control, balance, and movement; nevertheless, it may also induce a diverse array of impacts on sensory perception, cognitive function, mental health, and other areas [1]–[3]. PD leads to the deterioration of a specific region in the brain, known as the basal ganglia. As this region declines, patients lose the capabilities that those regions formerly had. Researchers have discovered that PD induces a significant alteration in brain chemistry. The human brain utilizes neurotransmitters to regulate communication among neurons; PD is characterized by insufficient levels of dopamine, a crucial neurotransmitter, as shown in Figure 1.

Neurodegenerative disorders impact millions globally, with Alzheimer's disease (AD) and PD being the most prevalent forms; also, the likelihood of developing these conditions significantly increases with advancing age. This necessitates enhancing the current methodologies to identify these disorders [4],[5]. Handwriting and hand sketching are the result of a complex interaction of cognitive, kinesthetic, and perceptual-motor abilities and are among common activities affected by neurological illnesses. A very notable example of standardized handwriting tests used to assist in diagnosing problems is the Minnesota Handwriting Assessment (MHA) [6]–[8]. The MHA is a clinical test based on handwriting analysis.

Handwriting attributes are readability, speed, shape, alignment, size, and spacing [6]. AD and PD substantially impact handwriting. Using digitizers has enabled quantitative hand-drawing assessments to provide insights into motor deficits associated with PD. Nonetheless, the diminished naturalness of the gesture and the inadequate user-friendliness of data collection block the integration of such technologies into clinical practice. To address these drawbacks, researchers developed an innovative smart ink pen to evaluate spiral drawings to enhance the characterization of motor symptoms associated with PD. The device, used on paper like a conventional pen, is augmented with motion and force sensors.

As time progresses and technology advances, the emergence of artificial intelligence (AI) has introduced innovative possibilities for healthcare, especially in illness detection and treatment. Utilizing AI methods may improve early detection and diagnostic deficiencies. Researchers have attempted to use ML methods to accurately and speedily detect PD patients through magnetic resonance imaging (MRI) and positron emission tomography (PET) [9]–[11]. One of the first indicators of PD is compromised motor control, especially in fine motor abilities. Individuals with PD often encounter tremors, difficulty coordinating fine motor movements, and diminished accuracy in manual activities. These deficits present in several forms, including handwriting and drawing activities. For instance, people may display micrographia (writing that gradually diminishes in size) or have difficulties executing basic designs, such as spirals or circles. Using hand-drawn patterns for PD detection is grounded in the evident correlation between motor impairment and drawing ability. Analyzing an individual's execution of a given activity, such as drawing a spiral, might reveal modest motor anomalies before the manifestation of more severe symptoms.

### 1.1. Objective

- To find appropriate AI models for diagnosing PD using different styles of handwriting and hand-drawing.
- To compare the transfer learning model and the transformer model for the classification of PD.
- To develop the HwdPD framework for diagnosing PD based on hand-drawing and handwriting tests, including Arabic handwriting.
- To develop a mobile application for diagnosing PD.

### 1.2. Research question

- What are the appropriate AI models that can help detect PD?
- How can the transformer model achieve high performance compared to DL models?
- How does the system diagnose PD?
- How do we develop a mobile application to diagnose PD?

### 1.3. Contribution

Due to the severity of PD, diagnosing it at an early stage is key. The proposed HwdPD framework uses hand-drawing and Arabic handwriting samples to analyze and predict PD. This framework was developed using VGG19 and transformer models.

- In this research, we developed a framework based on DL and a transformer for diagnosing PD using patients' hand movements.
- The HwdPD framework shows high accuracy for detecting PD based on hand-drawing and handwriting, including Arabic handwriting.
- In the proposed research, we have developed a mobile application to help PD patients and early PD diagnosis by doctors.

## 2. Study background

Currently, there are no accurate biomarkers for PD, making clinical diagnosis difficult [8]. Additionally, doctors' subjective awareness significantly impacts PD evaluation. Accelerometers, electromyography, and laser displacement sensors are examples of motion-takeover equipment that can measure tremor quantitatively [12].

Authors in [13] used visuals derived from time series analysis of sketching assignments. The tablet's raw signals were converted into matrices, and the output of this device was processed by a convolutional neural network (CNN) architecture. A CNN pre-trained on the ImageNet dataset function was used to analyze the PD images. Using spectral representations generated from tablet data, another work modeled tremor and other symptoms [14]. PD images were generated by stacking these signal representations, and a CNN model with 1 × 5 and 1 × 3 kernel sizes was fed into the model. Authors in [15] used an ML approach to examine drawings from three datasets (PaHaW [16], HandPD [17], and NewHandPD [18]) and applied the AlexNet model based on ImageNet and data augmentation approaches for enhancing the proposed systems for detecting PD. Authors in [19] built a CNN using a pooling layer, three convolutional layers, and two blocks. Similarly, the authors of [20] used pre-trained models built on ImageNet to enhance the model's generalizability. Models have also been pre-trained using datasets such as MNIST and UJIpenchars, which have semantic similarities with writing tasks.

Nayan et al. [21] diagnosed PD based on the disruption of olfactory loss and rapid eye movement sleep behavior. This research used contemporary ML techniques, including boosted logistic regression, RF, Bayesian networks, and multilayer perceptrons. The boosted LR model demonstrated an accuracy of 97%, suggesting its potential use for early prediction of PD. Senturk et al. [22] introduced an ML-based approach for diagnosing PD via feature selection and classification. Techniques for feature selection include recursive feature elimination and feature importance assessment. Compared to classification trees, neural networks, and support vector machines (SVMs), the study revealed that SVM using a Rotation Forest Ensemble (RFE) had superior performance. With an emphasis on voice features, the subsequent PD diagnosis had a precision of 93.84%. Polat et al. [23] proposed ML techniques for identifying PD using speech signals. SMOTE preprocessing was applied to the dataset.

Pereira et al. [24] developed a handwriting technology-based PD detection technique. They proposed a CNN architecture based on features that might be extracted from smart pen signals. Optimum Path Forest (OPF) achieved the greatest accuracy for meanders and spirals (more than 83%). This technique discovered writing issues connected to PD through the use of pen-based signals. Taleb et al. [25] built CNN and CNN-BiLSTM models for time series classification to detect PD. Spectrograms encoded with pen-based signals were used as input rather than raw time series data, generating CNN images. This system outperformed pre-engineered models; this research demonstrated that DL models may still be successful with insufficient data. Xu et al. [26] used an RF approach to differentiate between PD and health controls (HC) patients using handwriting data, integrating RF classifiers with PCA. They built six distinct RF models for handwritten tests to produce class probability vectors that reflect individual category predictions. The final prediction was determined using a voting technique. They used stratified k-fold cross-validation to demonstrate that their ensemble model outperformed individual RF-based techniques on six handwriting tests. The RF ensemble model demonstrated encouraging results across a range of handwriting evaluation metrics, including accuracy (89%). Regarding classification results, their method performed better than conventional ML techniques, such as SVM. Peter et al. [16] used ML approaches for classifying handwriting samples from 38 healthy and 37 PD patients. This research analyzed novel pressure aspects based on the pressure exerted on the writing surface. The three classifiers used to distinguish PD patients and HC were KNN, ensemble Adaptive Boosting, and SVM. Classifying PD using handwriting kinematics and pressure was best accomplished by SVM, which achieved an accuracy of 81%. Table 1 summarizes the existing studies regarding PD using various AI algorithms.

**Table 1.** Existing systems of PD diagnosis using AI algorithms.

Ref	Year	Model	Domain name	ACC%
Ref [27]	2020	CNN	EEG dataset	88.25
Ref [22]	2020	Various ML models	Voice_PD dataset	91
Ref [28]	2020	CNN	Speech language	99.3
Ref [29]	2021	KNN, SVM	Biomedical record PD	94.7
Ref [30]	2019	FCM	Voice PD	68
Ref [31]	2012	Fuzzy, neural network	Gait	77.33
Ref [32]	2014	MCNN	Gene expression	95.55
Ref [33]	2019	McRBFN	MRI image	87.21
Ref [34]	2019	DL, RNN	PD handwriting	89.64
Ref [35]	2019	CNN, SVM	PD handwriting	86.67
Ref [24]	2016	CNN	PD hand-drawing	98
Ref [6]	2020	AlexNet	PD hand-drawing	89
Ref [36]	2019	Adaboost	PD hand-drawing	76.44
Ref [15]	2021	Transfer learning	PD hand-drawing and handwriting	99.22
Ref [37]	2018	KNN, DT	PD hand-drawing	92.19

## 3. Materials and methods

**Figure 2.**
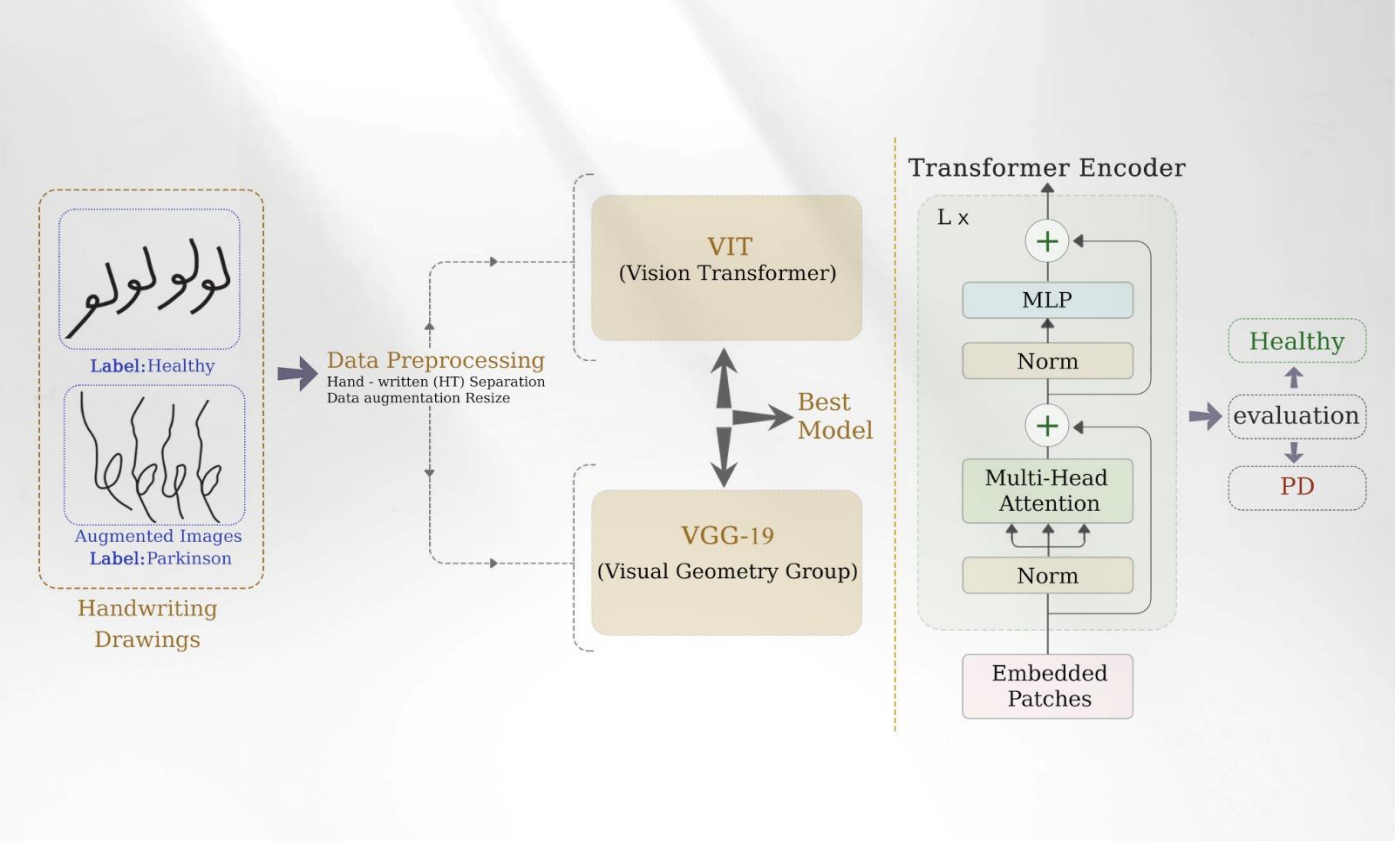
HwdPD framework.

**Figure 3.**
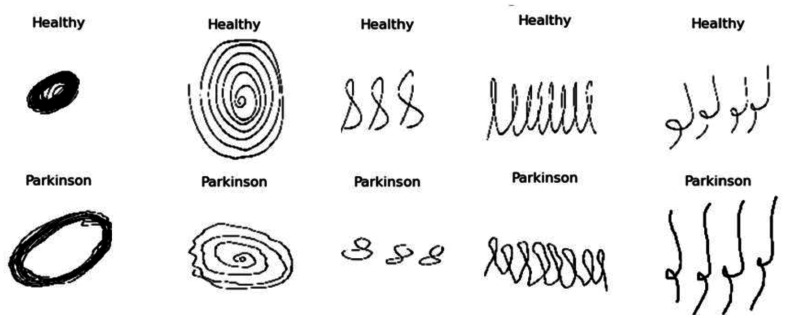
Samples of the proposed dataset for developing our system.

Figure 2 illustrates the HwdPD framework for diagnosing PD by analyzing patient handwriting and hand-drawing samples. This system utilizes Arabic handwriting samples, namely “لو”, and English handwriting, namely “eight”, and standard hand-drawings, such as “spiral”, “wave”, and “ellipse”, as shown in Figure 3. The dataset was collected from [38]. This HwdPD framework was developed using VGG19 and transformer models to detect PD.

### 3.1. Dataset

The HwdPD framework was examined using standard author data [38]. This was the second study after the authors who developed the dataset; we used the dataset to improve the existing system [38]. The dataset was collected from PD patients as hand-drawings and Arabic handwriting samples from the Neurology Department, Habib Bourguiba Hospital, Sfax, Tunisia. The dataset comprises 63 images of each class (i.e., spiral, wave, and ellipse, as well as Arabic handwriting for eight and hello “لو”) of 30 PD patients and 33 healthy individuals. The dataset comprised female and male patients, with an average age of 58 years.

### 3.2. Dataset augmentation

An augmentation technique was employed to improve the dataset, utilizing rotation, translation, blurring, brightness modification, scaling with factors below and above one, and other conventional methods to enhance image collections. Rotation was the only augmentation approach that was deemed appropriate. Despite the lack of attention to scale, the dimensions of the drawn spiral, wave, and ellipse could provide information about the target condition; for example, many studies have shown that micrographia, characterized by smaller and more compact waves and spirals, is typically produced by people with PD. Since both feature extraction processes work on the masked image, removing the original brightness, blurring, and brightening was rejected, since they might hide important information inside the image.

$${\text{A}}^{\prime }=\text{A}*\text{cos}\left(\theta \right)-\text{B}*\text{sin}\left(\theta \right)$$ (1)

$${\text{B}}^{\prime }=\text{A}*\text{sin}\left(\theta \right)+\text{B}*\text{cos}\left(\theta \right)$$ (2)

Where A, A′, and B, B′ are round points, A is the pixel's horizontal coordinate, measured in pixels from the left edge, and B is the pixel's vertical coordinate, measured from the top edge.

$${\text{X}}^{\prime }=\text{X}+\text{TX}$$ (3)

$${\text{y}}^{\prime }=\text{y}+\text{Ty}$$ (4)

where x, y represent the original coordinates of a pixel in the image, x′, y′ indicates the coordinates after translation.

$$x^{\prime }=sx*x$$ (5)

$$y^{\prime }=sy*y$$ (6)

Where x′, y′ are the new coordinates after shearing values, the and are vertically and horizontally shear factors.

$$x^{\prime }=x+\lambda *y$$ (7)

$$y^{\prime }=y$$ (8)

**Figure 4.**
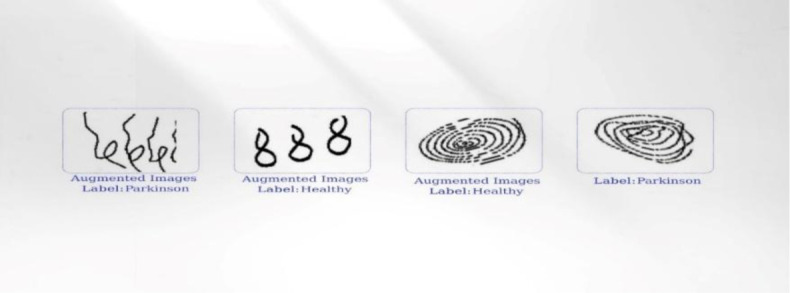
PD images after augmentation preprocessing.

Using the augmentation method, the images were scaled to 100 × 100 pixels and then transformed into NumPy arrays using the function to enable tensor computations. Stored in a label array, the relevant class labels were manually assigned as integers, 0 for healthy and 1 for PD. Both image data arrays were normalized by dividing pixel values by 255.0, hence transforming them from the range [0, 255] to [0, 1], which is necessary for consistent gradient descent training, after data loading. A real-time data augmentation generator was applied after splitting the dataset exclusively to the training set, adding random transformations such as rotations, translations, shearing, zooming, and horizontal flipping. Simulating additional versions of the original data without expanding the dataset size provides the model with better generalization. Figure 4 shows a series of images produced by rotating at different angles to show the augmentation process.

### 3.3. VGG 19 model

**Figure 5.**
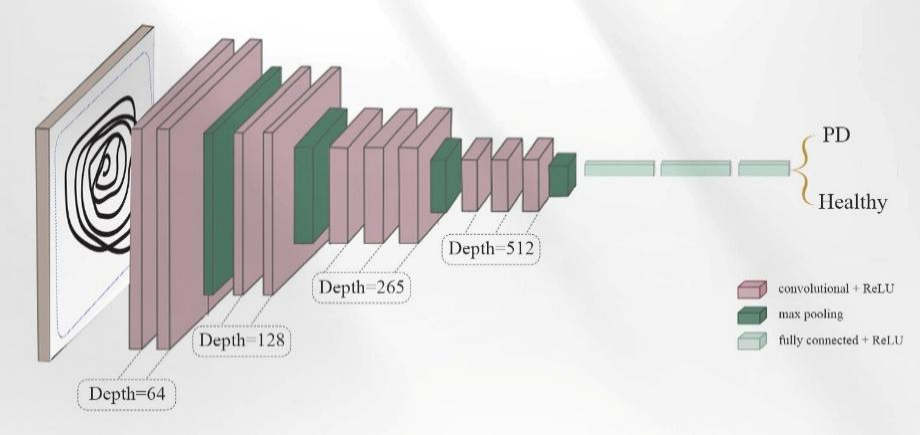
VGG19 of the HwdPD framework.

The VGG19 network is a type of AlexNet architecture, featuring sequential convolutional layers that utilize increasing filters at deeper levels of the network. Figure 5 displays the architecture of VGG19 to detect PD.

F=conv(x,W)+b (9)

where the *f* denote output of the HwdPD framework, and *x* denote the input of training dataset, and *b* is the basis for justifying the *w* weights of the neural network.

ReLU(z)=max(0,z) (10)

$$y=W_{f}.x+b_{f}$$ (11)

Where *w* is the weight of the neural network of FC, y is the output of PD, health is a feature map to FC, and *x* is a PD image.

$$y_{i}=\frac{e^{z_{i}}}{\sum_{j}e^{z_{i}}}$$ (12)

where *z_i_* is the predicted score for class of dataset PD or HC, and *y_i_* is the output probability after normalization.

The VGG19 architecture, enhanced with custom classification layers, was used to categorize sample images as either healthy or indicative of PD. The architecture initiates with an input layer designed for 100 × 100 images, followed by the VGG19 convolutional base, which consists of 16 convolutional layers organized into 5 blocks, each concluding with a MaxPooling2D layer to diminish spatial dimensions. The architecture comprises two convolutional layers with 64 filters in Block 1, 2 layers with 128 filters in Block 2, 4 layers with 256 filters in Block 3, 4 layers with 512 filters in Block 4, and 4 layers with 512 filters in Block 5. After the final pooling layer, a global average pooling layer transforms the output from dimensions (3, 3, 512) into a one-dimensional vector of size 512. A custom dense layer comprising 64 units with ReLU activation is incorporated, followed by a final dense output layer featuring two units with softmax activation for binary classification. The VGG19 base is totally frozen, rendering it non-trainable; consequently, only the appended dense layers undergo updates during training. The model comprises 20,060,546 parameters, with only 32,962 being trainable. Table 2 shows the VGG19 parameters of the HwdPD framework.

**Table 2.** VGG19 parameters of HwdPD framework.

Layer (type)	Output shape	Parameter #
input_2 (InputLayer	(100,100,3)	0
block1_conv1 (Conv2D)	(100,100,64)	1792
block1_conv2 (Conv2D)		36928
block1_pool (MaxPooling2D)	(50,50,64)	0
block2_conv1 (Conv2D)	(50,50,128)	73856
block2_conv2 (Conv2D)		147584
block2_pool (MaxPooling2D)	(25,25,128)	0
block3_conv1 (Conv2D)	(None, 25, 25, 256)	295168
block3_conv2 (Conv2D)		590080
block3_conv3 (Conv2D)		
block3_conv4 (Conv2D)		
block3_pool (MaxPooling2D)	(None, 12, 12, 256)	1
block4_conv1 (Conv2D)	(None, 12, 12, 512)	1180160
block4_conv2 (Conv2D)		2359808
block4_conv3 (Conv2D)		
block4_conv4 (Conv2D)		
block4_pool (MaxPooling2D)	(None, 6, 6, 512)	0
block5_conv1 (Conv2D)	(None, 6, 6, 512)	2359808
block5_conv2 (Conv2D)		
block5_conv3 (Conv2D)	(None, 6, 6, 512)	2359808
block5_conv4 (Conv2D)	(None, 6, 6, 512)	2359808
block5_pool	(None, 3, 3, 512)	0
block5_conv2	(None, 6, 6, 512)	2359808
global_average_pooling2d lobalAveragePooling	(None, 512)	0
dense (Dense)	(None, 2)	130

### 3.4. Vision transformer (ViT)

**Figure 6.**
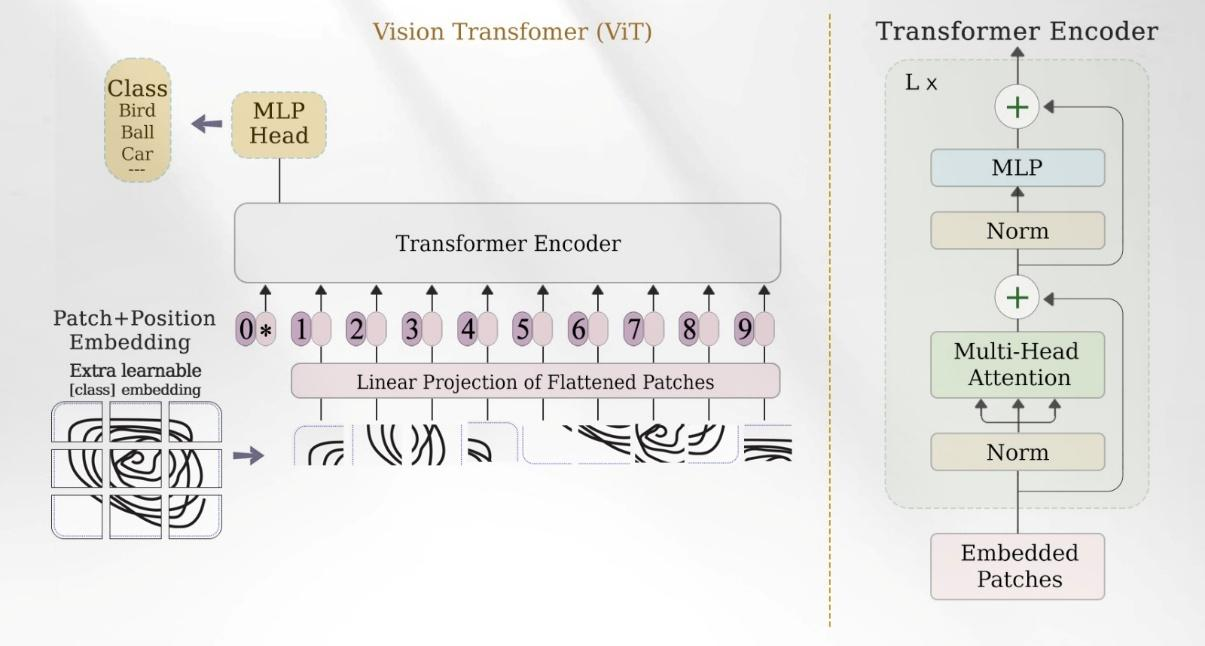
ViT model based on the HwdPD framework.

The transformer architecture employs a self-attention mechanism that functions as a fundamental layer in transformer network architecture, designed to address complex issues in computer vision. In contrast to RNNs, it removes reliance on prior states, enabling transformers to analyze sequence components concurrently and markedly improve computing performance. The Vision transformer (ViT-B16) model used here analyzes 128 × 128 pictures by segmenting them into 16 × 16 patches and embedding them for transformer-based categorization. The design comprises a patch embedding layer (Conv2D), positional embedding, and 12 transformer encoder blocks, each tasked with learning contextual associations via multi-head self-attention and feedforward layers. The conclusive categorization is executed using an MLP head with a softmax activation function for two output categories: “healthy” and “parkinson”. The architecture of the ViT-B16 model is presented in Figure 6. The parameters of the ViT model are presented in Table 3.

Algorithm ViT:

Let's input $\text{x}\in {\text{R}}^{\text{AxBxWxC}}$, where A is the number of PD images, represent the spatial dimensions (height and width) of each image, and CC is the number of channels.

1. PD image dimensional

Each PD image ${\text{X}}_{\text{i}}\in \text{R}$ with label ${\text{y}}_{\text{i}}\in \{\text{PD and healthy}\}$

Normalization

$${\text{x}}_{\text{i}}\to \frac{{\text{x}}_{\text{i}}}{255}$$ (13)

Using one-hot encoding for converting the y_i_ class label into {0,1}, where 0 represents PD and 1 represents healthy

Augmentation

$${\text{x}}_{\text{i}}=\text{T}({\text{X}}_{\text{i}},\text{θ)}$$ (14)

Where θ is the rotation angle and transformation, where θ is the rotation angle and transformation in the standard two-dimensional Cartesian coordinate system, using the basis vectors (1,0) and (0,1).

2. Patching

Dividing each PD image into RxK where K=16

$${\text{N}}_{\text{K}}=\frac{\text{BxW}}{{\text{K}}^{2}}=\frac{128\text{x}128}{16^{2}}=64$$ (15)

The patching is flattened into this vector x_ij_

$${\text{M}}_{\text{ij}}={\text{W}}_{\text{e}}{\text{x}}_{\text{ij}}+{\text{j}}_{\text{e}}$$ (16)

${\text{W}}_{\text{e}}\in {\text{R}}^{\text{Dx}({\text{k}}^{2}.\text{C})}$ embeds a weighted matrix of D and ${\text{M}}_{\text{ij}}\in {\text{R}}^{\text{D}}$ representation of the patch embedded, the encoding of the patch is represented by ${\text{j}}_{\text{e}}\in {\text{R}}^{\text{D}}$

Label taken from PD class ${\text{M}}_{\text{i}0}\in {\text{R}}^{\text{D}}$ is indicated by sequences

$${\text{M}}_{\text{i}}=[{\text{M}}_{\text{i},0},{\text{M}}_{\text{i},1}\ldots \ldots \ldots \ldots \ldots {\text{M}}_{\text{i},}{\text{N}}_{\text{p}}]\in \in {\text{R}}^{\text{Dx}({\text{N}}_{\text{p}}+1)\text{XD}}$$ (17)

3. Encoder of the transformer

The ViT-B16 model consists of L = 12 encoder

Normalization

$${\overset{`}{\text{Nor}}}_{\text{i}}=\text{LN}\left({\text{Nor}}_{\text{i}}\right)\frac{{\text{Nor}}_{\text{i}-\text{µ}}}{\sqrt{{\text{σ}}^{2}}+\varepsilon }.\text{γ}+\text{β}$$ (18)

Where µ,σ is the mean and variance for the normalization of images, and γ,β is the parameter.

4. Multi-head self-attention

$$\text{Attention}({\text{Q}}_{\text{h}},{\text{F}}_{\text{h}},{\text{V}}_{\text{h}})=\text{softmax}\left(\frac{{\text{Q}}_{\text{h}},{\text{F}}_{\text{h}}^{\text{T}}}{\sqrt{{\text{d}}_{\text{k}}}}\right){\text{V}}_{\text{h}}$$ (19)

$${\text{Q}}_{\text{h}}=\overset{`}{\text{Nor}}{\text{W}}_{\text{h}}^{\text{Q}},{\text{F}}_{\text{h}}=\overset{`}{\text{Nor}}{\text{W}}_{\text{h}}^{\text{F}},{\text{V}}_{\text{h}}=\overset{`}{\text{Nor}}{\text{W}}_{\text{h}}^{\text{V}}$$ (20)

$${\text{W}}_{\text{h}}^{\text{Q}},{\text{W}}_{\text{h}}^{\text{F}},{\text{W}}_{\text{h}}^{\text{V}}\in {\mathbb{R}}^{{\text{Dxd}}_{\text{k}}}=\text{D}/\text{B}=64$$ (21)

$$\text{MSA}\left(\text{Nor}\right)=\text{Concation}\left(\text{head}1`\ldots \ldots \ldots \ldots \ldots \ldots \ldots \text{HeadB}\right){\text{W}}_{0}\text{ and }{\text{W}}_{0}\in {\mathbb{R}}^{DxD}$$ (22)

$${\text{Nor}}^{\prime \prime }=\text{MSA}({{\text{Nor}}^{\prime }}_{\text{i}})+{\text{Nor}}^{\prime }$$ (23)

$$\text{FNN}\left(\text{z}\right)={\text{W}}_{2}.\text{GELU}\left({\text{W}}_{1\text{z}}+{\text{b}}_{1}\right)+{\text{b}}_{2}$$ (24)

$$\text{Where }{\text{W}}_{1}\in \mathbb{R}{\text{D}}_{\text{ff}}\text{xD},{\text{W}}_{2}\in \mathbb{R}{\text{D}}_{\text{ff}}\text{xD},{\text{W}}_{2}=3072$$ (25)

$${\text{z}}_{\text{i}}=\text{FNN}\left(\text{LN}\left({{\text{Nor}}^{\prime \prime }}_{\text{i}}\right)\right)+{{\text{Nor}}^{\prime \prime }}_{\text{i}}$$ (26)

$${\hat{\text{y}}}_{\text{i}}=\text{softmax}({\text{W}}_{{\text{cz}}_{\text{i},0}}+{\text{b}}_{\text{c}})$$ (27)

**Table 3.** Parameters of the ViT model.

Hyperparameter	Value
ViT model_ Optimizer	Adam
ViT model_Learning Rate	0.0001
Batch size	8
Epochs	20
Image size	128 × 128

## 4. Experimental results and discussion

The experiment used the Keras deep learning and transformer framework in Python, executed on the Kaggle cloud with a GPU P100. The operating environment consisted of Windows 10, with 16 GB of RAM, a 9th-generation Core i7 processor, and software implementation. The HwdPD framework was built with the VGG19 and ViT-6 models. This study used several forms of PD handwriting and hand-drawing samples to diagnose PD, including 63 spiral images, 63 waves, 63 ellipses, 63 “eights”, and 63 “لو” from PD and healthy individuals. The framework has been validated in improving patients' mental health by many physicians, including those using physical assessments. The dataset was split between 15% for testing, 15% for validation, and 70% for training.

Evaluation metrics were used to evaluate the performance of the HwdPD framework. For binary classification, these were calculated as follows:

$$\text{TPR}=\frac{\text{TP}}{\text{TP}+\text{FN}}$$ (28)

$$\text{FPR}=\frac{\text{FP}}{\text{FP}+\text{TN}}$$ (29)

$$\text{F1-Score}=2\times \frac{\text{Precision}\times \text{Recall}}{\text{Precision}+\text{Recall}}$$ (30)

$$Recall=\frac{\text{True Positive}}{\text{True Positive}+\text{False Positive}}$$ (31)

$$\text{Precision}=\frac{\text{True Postive}}{\text{True Positive}+\text{False Negative}}$$ (32)

$$\text{Accuracy}=\frac{\text{TP}+\text{TN}}{\text{TP}+\text{TN}+\text{FP}+\text{FN}}$$ (33)

### 4.1. Experiment 1: Spiral images

**Figure 7.**
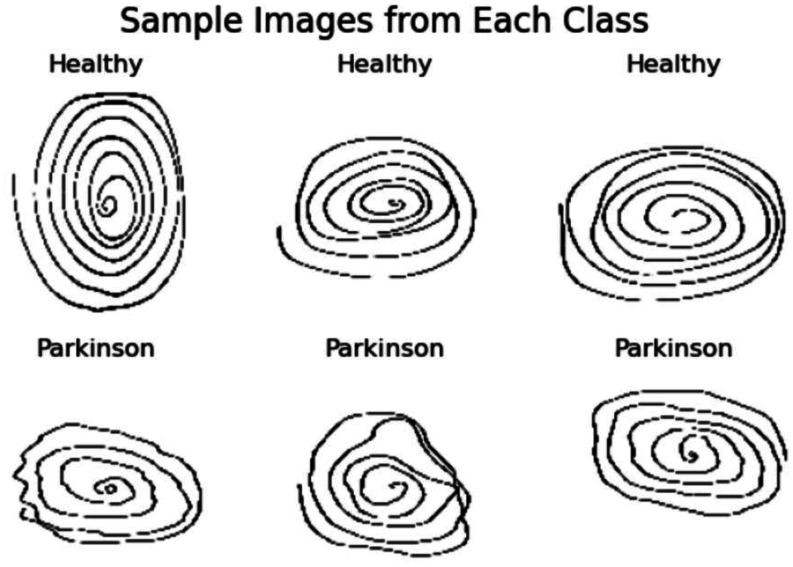
Samples of spiral images.

In this experiment, spiral images were used to detect PD. Figure 7 shows a spiral image sample collected from patients with PD and healthy individuals.

Table 4 shows the findings of the VGG19 and ViT models for detecting PD. The ViT and VGG19 models showed better accuracy in identifying PD using spiral images. VGG19 achieved 100% for both metrics in the healthy class. In the Parkinson class, it achieved 100% for both metrics. The ViT model showed 100% accuracy, with a high percentage for the health class recall (100%) and Parkinson class precision (100).

**Table 4.** Results of the HwdPD framework using spiral images.

VGG19 model
Class name	Precision (%)	Recall (%)	F1 score (%)	Support
Healthy [0]	100	100	100	
Parkinson [1]	100	100	100	
Accuracy	100
Macro Avg	100	100	100	10
ViT model
Class name	Precision (%)	Recall (%)	F1 score (%)	Support
Healthy [0]	100	100	100	
Parkinson [1]	100	100	100	10
Accuracy	100
Macro Avg	100	100	100	

**Figure 8.**
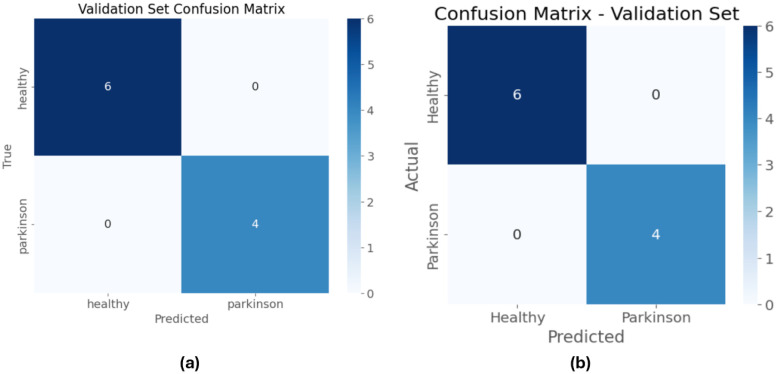
Confusion matrix of the HwdPD framework. a) VGG19 and b) ViT models.

Figure 8 depicts the confusion matrices of the HwdPD framework [VGG19 (a) and ViT (b)] regarding a binary classification task aimed at distinguishing between “healthy” and “Parkinson” samples. In the VGG19 model, six out of six healthy instances were correctly classified; four Parkinson instances were correctly classified. The ViT model demonstrated flawless classification performance, accurately identifying all six instances as healthy and the correct four as PD in both classifications, resulting in 100% accuracy, precision, and recall.

**Figure 9.**
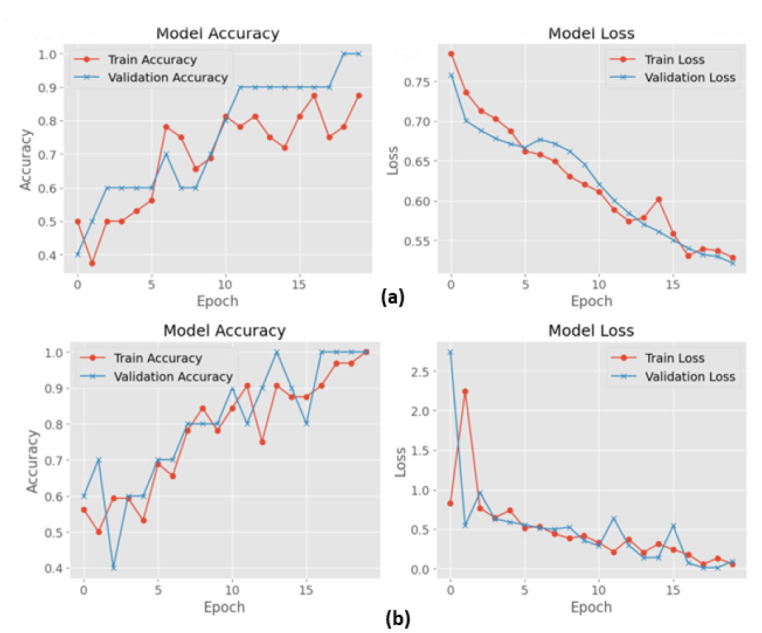
Performance of the HwdPD framework. a) VGG19 and b) ViT models.

Figure 9 displays the system accuracy as a function of time utilizing spiral images. On the y-axis, the percentage of data that was categorized properly is plotted. A useful measure of how well the training system was working is how well the validation system has performed. We noticed a disruption in the optimization process, which resulted in an improvement in accuracy to 50 epochs, a notable achievement. The VGG19 shows performance accuracy ranging from 50% to 100%, the ViT model attained a training accuracy of 100%, and the VGG19 and ViT models' loss reached 0.00.

### 4.2. Experiment 2: Wave images

**Figure 10.**
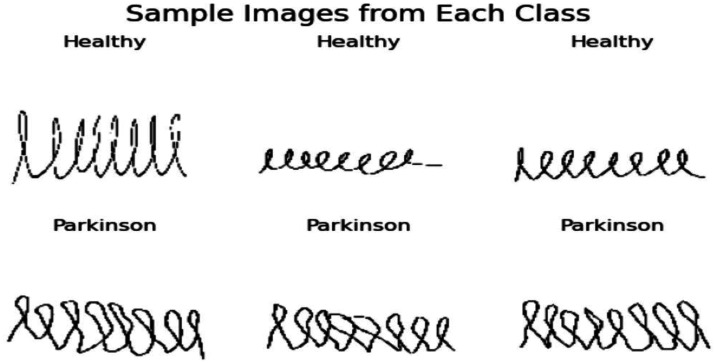
Samples of wave images.

As shown in Figure 10, in this experiment, we used hand-drawn wave images to test the proposed framework for detecting PD, comprising approximately 60 images from both PD and healthy subjects.

Table 5 shows the results of the VGG19 and ViT models using wave images for detecting PD. The VGG19 model achieved a higher precision for the healthy class (80%) but a lower recall (67%), indicating that it identifies most healthy instances while generating more false positives. Regarding Parkinson's disease, it demonstrates good recall (75%) but poor precision (60%), indicating that it accurately classifies cases with great confidence but fails to detect many actual instances. The total accuracy of VGG19 was 70%. Conversely, the ViT model exhibited superior performance, achieving an accuracy of 100%. It achieved excellent accuracy for healthy individuals (100%) but exhibited inferior recall (100%). In contrast, for Parkinson's patients, it demonstrated strong recall (100%) and precision (100%). This indicates that the ViT model shows superior efficacy in accurately identifying PD. Finally, ViT demonstrates superior overall performance, particularly for Parkinson's detection.

**Table 5.** Results of the HwdPD framework using wave images.

VGG19 model
Class name	Precision (%)	Recall (%)	F1 score (%)	Support
Healthy [0]	80	67	73	
Parkinson [1]	60	75	67	
Accuracy	70	
Macro Avg	70	71	70	10
ViT model
Class name	Precision (%)	Recall (%)	F1 score (%)	Support
Healthy [0]	100	100	100	2
Parkinson [1]	100	100	100	7
Accuracy	100
Macro Avg	100	100	100	

**Figure 11.**
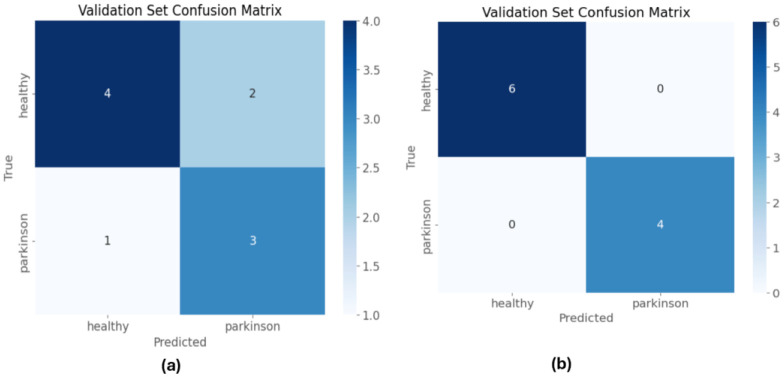
Confusion matrix of the HwdPD framework using wave images. a) VGG19 and b) ViT models.

The confusion matrices evaluate the performance of the HwdPD framework in classifying healthy patients and Parkinson's disease using wave images, as presented in Figure 11. The VGG19 model accurately classified four healthy and 3 PD cases in subplot (a). However, it misclassified two healthy cases as PD and one PD case as healthy. The ViT model in subplot (b) exhibits superior performance, accurately classifying all four PD and six healthy cases. Consequently, the ViT model demonstrates greater reliability, particularly in accurately identifying PD.

**Figure 12.**
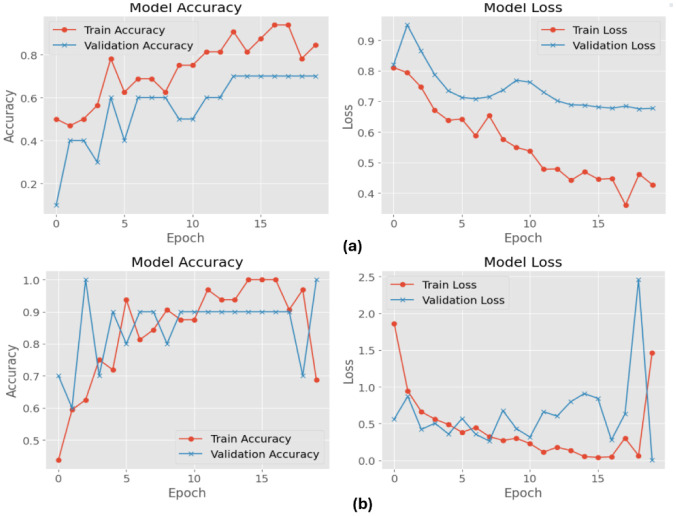
Performance of the HwdPD framework using wave images. a) VGG19 and b) ViT models.

Figure 12 illustrates the training and validation performance of the HwdPD framework using wave PD images. The VGG19 model started at 50% and reached 70%, whereas the test accuracy exhibited fluctuations but ultimately rose to a similar level by the conclusion of the training process. The ViT model's initial training and test accuracy were lower, approximately 43.75%, and exhibited greater variability; they ultimately stabilized, with training accuracy exceeding 100% at the training phase and test accuracy increasing to 85%.

### 4.3. Experiment 3: “Eight” images

**Figure 13.**
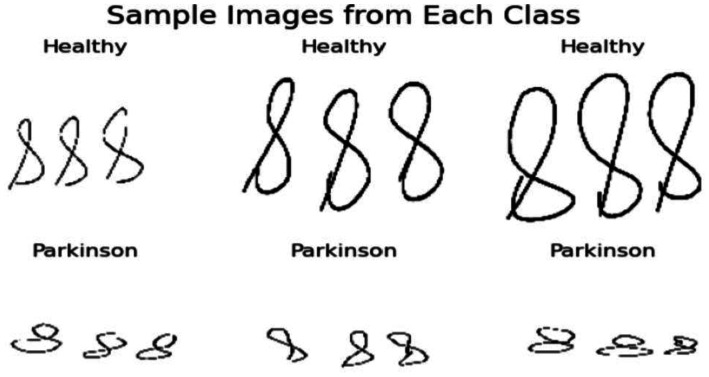
Samples of “eight” images.

The proposed framework used “eight” samples to detect PD. The number “eight” has edges, which can help identify PD. In this experiment, 60 “eight” images were analyzed by the VGG19 and ViT models. The sample PD images are shown in Figure 13.

Table 6 displays the classification metrics for identifying objects using HwdPD based on VGG19 and ViT models, with “eight” images. Employing VGG19, the model achieved an overall accuracy of 100% for both healthy and PD classes, with 100% precision, 100% recall, and an F1 score of 100%, signifying superior sensitivity in detecting PD. Both the ViT and VGG19 models scored the same percentage when using “eight” images.

**Table 6.** Results of the HwdPD framework using “eight” images.

VGG19 model
Class name	Precision (%)	Recall (%)	F1 score (%)	Support
Healthy [0]	100	100	100	
Parkinson [1]	100	100	100	
Accuracy	100
Macro Avg	100	100	100	10
ViT model
Class name	Precision (%)	Recall (%)	F1 score (%)	Support
Healthy [0]	100	100	100	
Parkinson [1]	100	100	100	
Accuracy	100
Macro Avg	100	100	100	10

**Figure 14.**
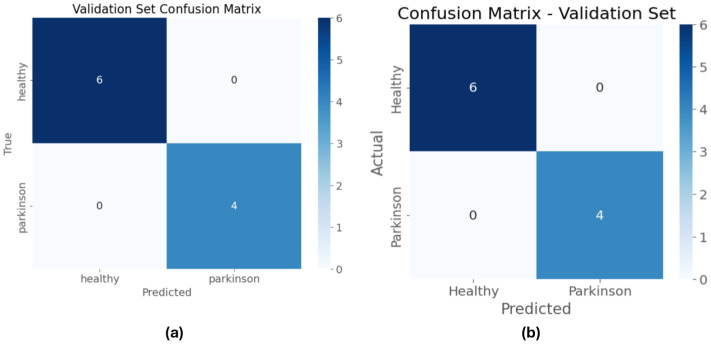
Confusion matrix of the HwdPD framework using “eight” images. a) VGG19 and b) ViT models.

Figure 14 displays the confusion matrices of the HwdPD framework, based on the VGG19 and ViT models, using “eight” images. Figure 14a shows the performance of VGG19 based on validation. VGG119 achieved perfect classification, correctly classifying six healthy cases and four PD cases. Both models made no false positives or false negatives, correctly differentiating between Parkinson's and healthy cases.

**Figure 15.**
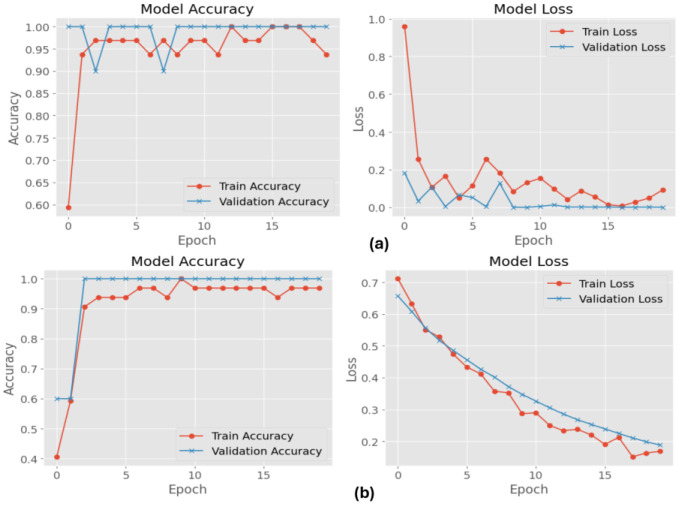
Performance of the HwdPD framework using “eight” images. a) VGG19 and b) ViT models.

Figure 15 demonstrates the performance across accuracy, loss, and classification metrics of the VGG19 and ViT models for detecting PD. The VGG19 model achieved an accuracy of 90%, which then increased to 100% in the validation phase. In the training phase, the VGG19 model started at 59% and reached 93%. ViT demonstrated good performance, ranging from 60% to 100%, and the loss of the ViT model reached 0.002.

### 4.4. Experiment 4: لو images

**Figure 16.**
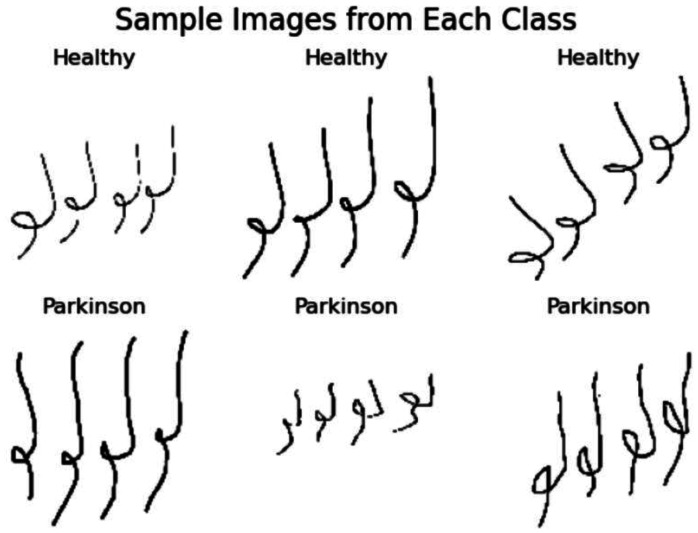
Samples of “لو” images.

In this experiment, we also tested the models using the Arabic handwriting “لو” to detect PD, as shown in Figure 16, as this word has a movement that can help detect the disease.

Table 7 indicates that the performance of the VGG19 and ViT models in detecting PD using the Arabic word “لو” is comparable. The VGG19 model achieved an accuracy of 75% for healthy patients, although it had a poor recall of 50%, indicating that it overlooked several healthy individuals. The VGG19 model performed well on Parkinson's patients, achieving a recall of 100% but a precision of 57%, and an overall accuracy of 70%. The ViT model achieved high precision and recall for both classes (precision: 100% for healthy, 100% for Parkinson; recall: 100% for healthy, 100% for Parkinson), resulting in an overall accuracy of 100%. The macro-average metric of the ViT model achieved 100%. The findings demonstrate that the ViT model outperforms the VGG-19 model in reliably distinguishing between PD and healthy individuals.

**Table 7.** Results of the HwdPD framework using “لو” images.

VGG19 model
Class name	Precision (%)	Recall (%)	F1 score (%)	Support
Healthy [0]	100	50	67	
Parkinson [1]	57	100	73	
Accuracy	70
Macro Avg	79	75	70	
ViT model
Class name	Precision (%)	Recall (%)	F1 score (%)	Support
Healthy [0]	100	100	100	
Parkinson [1]	100	100	100	
Accuracy	100
Macro Avg	100	100	100	

**Figure 17.**
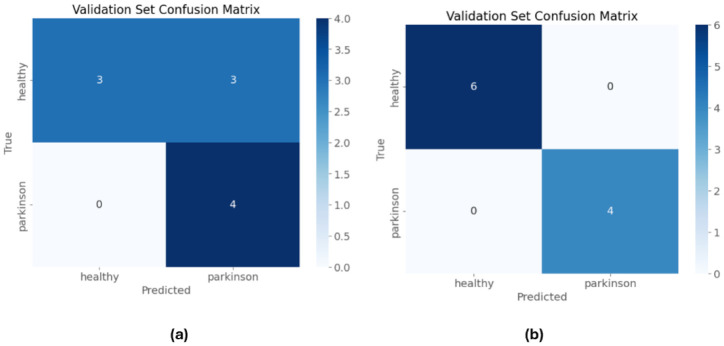
Confusion matrix of the HwdPD framework using “لو” images. a) VGG19 and b) ViT models.

Figure 17 presents the confusion matrices of the HwdPD framework based on the VGG19 and ViT models used for PD and healthy patients. The VGG19 matrix demonstrates that the four PD samples were correctly classified; however, in the healthy class, three out of six healthy patients were incorrectly diagnosed as having Parkinson (false negative). In opposition, the ViT model presents a more equitable approach, accurately identifying all six healthy instances and all four PD samples.

**Figure 18.**
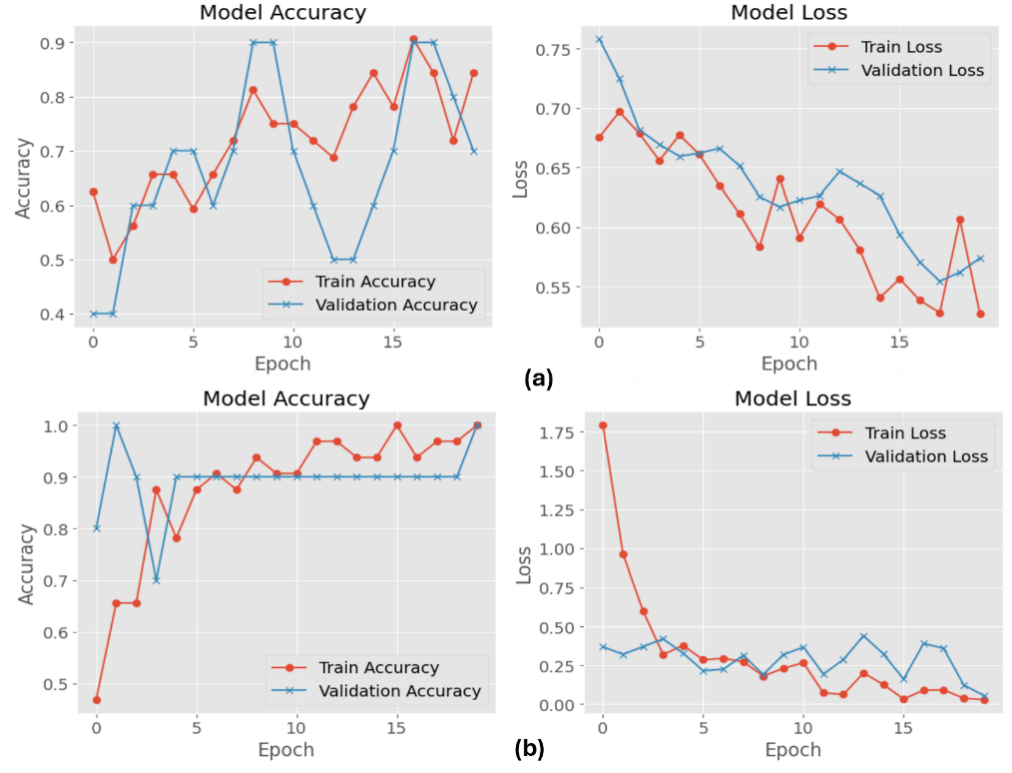
Performance of the HwdPD framework using “لو” images. a) VGG19 and b) ViT models.

Figure 18 presents the training and validation results for the VGG19 and ViT models. The first epoch of the VGG19 model achieved an accuracy of 70% with a loss of 0.54 in the training stage. However, validation accuracy was 70% with a loss of 0.53, with 15 epochs. The ViT model exhibited commendable accuracy and loss performance, achieving 100% accuracy during the validation phase and a loss of 0.00 across 20 epochs.

### 4.5. Experiments of HwdPD using ellipse images

**Figure 19.**
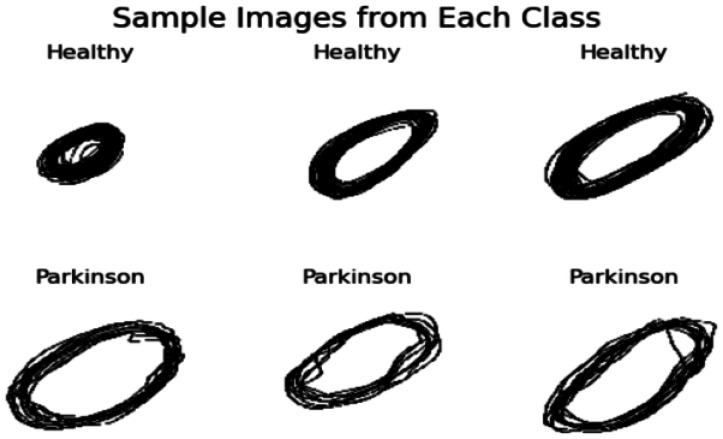
Samples of ellipse images.

This experiment used hand-drawn images of an ellipse to detect PD, as seen in Figure 19. Elliptical images are relevant in diagnosing PD, as patients with PD may make repeated attempts to draw them, which can aid in detecting the condition.

The detection results of PD using the HwdPD framework are shown in Table 8. For the healthy label, the VGG19 model obtained 100% accuracy and 83% recall. For the PD label, the precision was 80%, and recall was 100%. The VGG19 model, as a whole, was 92% accurate. The macro average of VGG19 scored 92% for recall and 90% for precision and F1 score. The ViT model demonstrated a high level of performance, achieving 100% precision, recall, and F1 score for both classes, along with an overall accuracy of 100%.

**Table 8.** Results of the HwdPD framework using ellipse images.

VGG19 model
Class name	Precision (%)	Recall (%)	F1 score (%)	Support
Healthy [0]	100	83	91	6
Parkinson [1]	80	100	89	
Accuracy	90
Macro Avg	90	92	90	
ViT model
Class name	Precision (%)	Recall (%)	F1 score (%)	Support
Healthy [0]	100	100	100	
Parkinson [1]	100	100	100	
Accuracy	100
Macro Avg	100	100	100	

**Figure 20.**
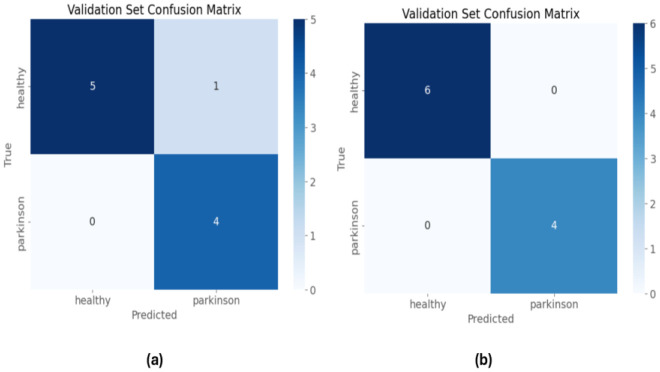
Confusion matrix of the HwdPD framework using ellipse images. a) VGG19 and b) ViT models.

Figure 20 presents confusion matrices of the HwdPD framework for classifying individuals as healthy or afflicted with PD. In subplot (a), the VGG19 model shows inaccurate classification, correctly identifying all PD samples (4/4) but misclassifying five out of six healthy individuals as having Parkinson's. In subplot (b), the ViT model demonstrates perfect classification accuracy, correctly identifying all six healthy and six PD without error.

**Figure 21.**
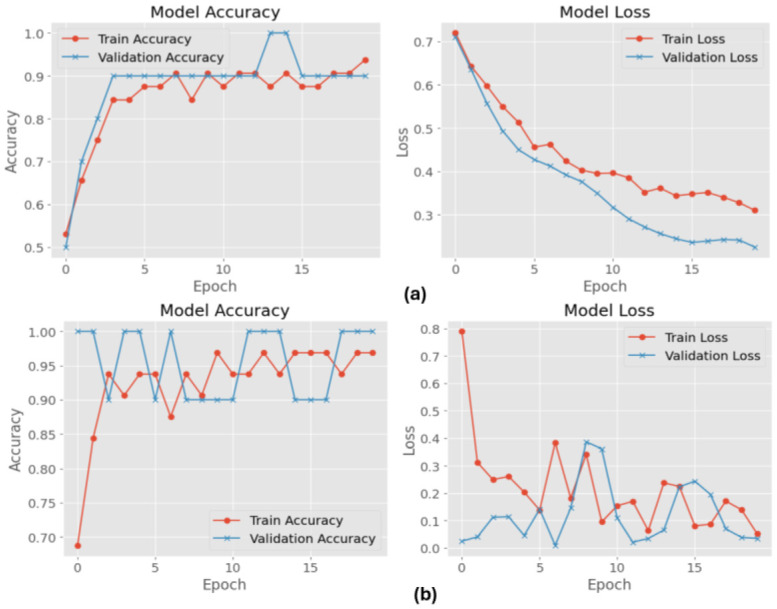
Performance of the HwdPD framework using ellipse images. a) VGG19 and b) ViT models.

Figure 21 displays the performance outcomes of HwdPD for diagnosing PD using ellipse images. The subplot (a) shows the VGG19 performance. The training accuracy exceeded 93.75%, and the validation accuracy was also 90%. On the other hand, the ViT model achieved 100% accuracy in both training and testing for detecting PD.

## 5. Model development

The development of the HwdPD framework based on AI systems in the healthcare sector is gaining increased attention from the scientific community. Datasets from patients and processes recorded in electronic forms may be processed and evaluated by AI systems, facilitating the development of more effective mental health conditional diagnosis and intervention methods. Examining hand-drawing and handwriting samples from individuals with PD has enhanced the comprehension of neural and motor mechanisms involved in pathological and physiological states. The HwdPD framework offers a non-invasive technique for measuring disease progression and, crucially, serves as a tool for early diagnosis.

**Figure 22.**
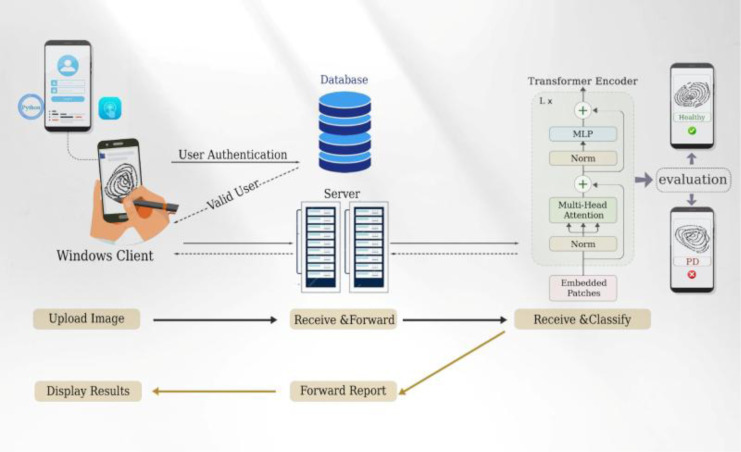
HwdPD deployment.

Figure 22 illustrates the deployment of the HwdPD framework for diagnosing PD via recording images collected from patients. This technique incorporates three forms of hand-drawn images: spiral, wave, and ellipse, as well as two styles of handwriting: “eight” and Arabic “لو”. This system, built using mobile architecture, assists detectors and patients in identifying PD at early stages. It is developed based on AI. During the deployment phase, it was observed that the system achieved high accuracy using the VGG19 and ViT models.

### 5.1. HwdPD system architecture

#### 5.1.1. Mobile application

This graphic user interface can help doctors and patients use it efficiently. The interface allows users to upload a handwriting or hand-drawing sample.

#### 5.1.2. User authentication

The client interacts with a MySQL Database to authenticate users. Access to the system's features is restricted to verified users only.

#### 5.1.3. MySQL database

Utilized for user authentication and the storage of user credentials. Upon entering the correct login credentials, the server verifies the user.

#### 5.1.4. Server

The server works as middleware between the client, the database, and the HwdPD. It sends the submitted images to the proposed HwdPD framework and obtains a classification of the images as either “PD” or “healthy”.

#### 5.1.5. Transformer model

The model analyzes the hand-drawing or handwriting samples and classifies these images as “PD” or “healthy”.

### 5.2. Preprocessing steps

#### 5.2.1. Upload images

This dialog can help patients draw and write images.

#### 5.2.2. Receive and forward (server)

The server can receive and forward images to a transformer model.

#### 5.2.3. Forward report

JSON format is used to forward the classification results to clients.

#### 5.2.4. Display results

This dialog shows the results for patients and doctors (“healthy” or “PD”).

This study was compared with that of [38], who used the same dataset. Table 9 indicates that the HwdPD framework demonstrates excellent accuracy for hand-drawn and handwritten images.

**Table 9.** Comparison of our results with those of [38].

Types of images	Existing system	Proposed system
Spiral image	89.99	100
Wave image	89.99	100
Eight image	86.00	100
Ellipse image	93.33	100
لو image	80.00	92

## 6. Conclusion

PD is among the most prevalent neurological conditions. Diagnosing PD is essential for tracking the course of the disorder. Hand-drawn and handwritten samples are one of the clinical datasets that may assist in diagnosing PD. This research used a standardized dataset including hand-drawn elements, such as spirals, waves, and ellipses, and handwritten samples, such as “eight” and “لو”. The collection comprises 63 hand-drawing and handwriting samples: 33 from healthy patients and 30 from PD patients. In this study, the HwdPD framework was designed to detect PD. The primary objective of this system is to develop a mobile application that can assist doctors and patients in detecting PD at an early stage. During the development phase, we used two advanced models, namely VGG19 and ViT, to examine the HwdPD framework in identifying PD. It was observed that the system achieves excellent accuracy while using specific images. In this research, we emphasize that this HwdPD framework can serve as a diagnostic tool to assist healthcare officials and doctors in identifying PD early by utilizing straightforward methods that allow patients to express themselves through drawing and writing. The ViT model achieved a high percentage of correctly diagnosing PD using various types of drawing and handwriting images.

The AI-driven approach will generate a conclusive report on PD and health status. We believe that identification performance may be enhanced by leveraging further clinical information about PD. The HwdPD framework achieved state-of-the-art performance in various PD-related tasks using the original dataset developed for this task. The rapid progress in deep learning and transformer models and the increase in available data provide a chance to tackle these challenges imminently and facilitate the widespread use of this technology in healthcare environments.

## Use of AI tools declaration

The authors declare they have not used Artificial Intelligence (AI) tools in the creation of this article.
